# Implementing Australia's new guidelines for appropriate psychotropic medication use in residential aged care: Applying the Consolidated Framework For Implementation Research

**DOI:** 10.1111/ajag.70013

**Published:** 2025-03-14

**Authors:** Shakti Shrestha, Amanda J. Cross, Michelle Steeper, Angelita Martini, Dayna Cenin, Jertrude Smith, Francesca Glamorgan, Constance Dimity Pond, J. Simon Bell, Adam La Caze

**Affiliations:** ^1^ Faculty of Health, Medicine and Behavioural Sciences, School of Pharmacy and Pharmaceutical Sciences The University of Queensland, Dutton Park Campus Woolloongabba Queensland Australia; ^2^ Centre of Medicine Use and Safety, Faculty of Pharmacy and Pharmaceutical Sciences Monash University, Parkville Campus Parkville Victoria Australia; ^3^ Faculty of Medicine, Nursing and Health Sciences, School of Public Health and Preventive Medicine Monash University Melbourne Victoria Australia; ^4^ School of Allied Health The University of Western Australia Perth Western Australia Australia; ^5^ Calvary Health Care Sydney New South Wales Australia; ^6^ Brightwater Research Centre Brightwater Care Group Inglewood Western Australia Australia; ^7^ School of Population and Global Health The University of Western Australia Perth Western Australia Australia; ^8^ Lifeview Corporate Lifeview Pty Ltd. Wheelers Hill Victoria Australia; ^9^ Montefiore Randwick New South Wales Australia; ^10^ Wicking Dementia Research and Teaching Centre University of Tasmania Hobart Tasmania Australia

**Keywords:** dementia, guideline, implementation science, psychotropic drugs, residential aged care facility

## Abstract

**Objectives:**

Accumulating evidence about psychotropic medication‐related adverse events has had minimal apparent impact on overall rates of psychotropic medication use across Australian residential aged care facilities (RACFs). Australia's new Clinical Practice Guidelines for the Appropriate Use of Psychotropic Medications in People Living with Dementia and in Residential Aged Care were released in April 2023. This study aimed to identify contextual factors to inform strategies to implement the new Guidelines in Australian RACFs.

**Methods:**

A qualitative study using semi‐structured interviews was conducted with the participants representing four Australian residential aged care organisations. The interviews were recorded, transcribed verbatim, coded and thematically analysed. Factors were deductively coded using the Consolidated Framework for Implementation Research (CFIR) into three domains: outer setting, inner setting and individuals, which subsequently informed the fourth CFIR domain—implementation process.

**Results:**

Participants (*n* = 33) were aged care residents and their family members, occupational therapists, nurses, nurse practitioners, general practitioners, geriatricians and pharmacists. The outer setting factors included regulatory changes, increased workload and increased workforce demand. The inner setting factors were health digitalisation, governance and compliance culture. Individual factors included mindset towards psychotropics and staff capabilities. The implementation process domain comprised four key strategies—recognising workforce pressures, leveraging recent efforts, supporting local use of data and supporting team functioning.

**Conclusions:**

These outer setting, inner setting and individual factors represent an interconnected framework of potentially modifiable factors to guide the targeted implementation of Australia's new Guidelines. These four key strategies provide new approaches to support the translation of the Guidelines.

**Practice impact**

This article provides crucial insights for researchers, aged care provider organisations and policymakers to support aged care quality improvement practice through implementation strategies for the new Clinical Practice Guidelines for the Appropriate Use of Psychotropic Medications in People Living with Dementia and in Residential Aged Care.

## 1 INTRODUCTION

Psychotropic medications are frequently used among residents living with dementia and in aged care facilities. International studies suggest that one‐third of residents with dementia receive two or more psychotropic medications concurrently.^1^ United States data indicate that 80% of residents are prescribed psychotropic medications annually.^2^ An estimated 27% of European residents use antipsychotics and 40% use antidepressants.^3^ More than 21% of Australian residents are dispensed antipsychotics, 30% benzodiazepines and 37% antidepressants within 3 months of admission to an Australian residential aged care facility (RACF).^4^, ^5^, ^6^ Clinical practice guidelines recommend that psychotropic medications should not be used for changed behaviours without first trialling non‐pharmacological approaches for an adequate and agreed‐upon length of time and should only be administered as a last resort for the shortest use.^7^, ^8^, ^9^, ^10^ Nevertheless, there is evidence of over‐reliance on psychotropic medications,^11^, ^12^ despite accumulating evidence of harm and limited evidence of effectiveness.^11^, ^13^ Addressing over‐reliance on psychotropic medications for chemical restraint was one of the three issues prioritised for immediate action by the Australian Royal Commission into Aged Care Quality and Safety.^12^ In response, Australia's new Clinical Practice Guidelines for the Appropriate Use of Psychotropic Medications in People Living with Dementia and in Residential Aged Care (new Psychotropic Guidelines) were developed and released in April 2023.^7^

The translation and implementation of the new clinical practice guidelines into RACFs are dependent on a range of contextual factors, both internal and external, and include considerations of the people, processes and resources employed to ensure appropriate psychotropic medication use.^14^, ^15^ Contextual factors contribute to unexplained variation in psychotropic medication prescribing,^16^ and to the importance of organisational culture in determining patterns of appropriate medication use.^17^ Knowledge of contextual factors across the operational settings of RACFs can be used to frame implementation strategies for ensuring appropriate medication use. This is important because existing interventions to ensure appropriate use have had variable uptake, with just 21% of residents receiving a comprehensive multidisciplinary review of their medications within 3 months of RACF admission.^18^

This study explored RACF quality improvement processes for psychotropic medications through an implementation science framework to identify the contextual factors influencing guideline implementation.^19^ The Consolidated Framework for Implementation Research (CFIR), a widely accepted implementation science framework, has considerable use in aged care.^20^ CFIR provides a practical method for systematically identifying facilitators and barriers to implementation effectiveness and can inform implementation planning and adoption.^21^ This study aimed to identify contextual factors that may influence the implementation of the new Psychotropic Guidelines in residential aged care and inform implementation strategies.

## 2 METHODS

### 2.1 Study design and setting

This study was conducted in accordance with the COnsolidated criteria for REporting Qualitative research (COREQ) statement.^22^ Qualitative semi‐structured interviews were conducted with stakeholders across four Australian aged care provider organisations from New South Wales, Queensland, Victoria and Western Australia. The four organisations collectively operated 27 RACFs and were selected through convenience sampling. Australian aged care homes (RACFs or nursing homes) offer assisted living for older people who can no longer live at their home and need ongoing support with everyday tasks or health care.^23^, ^24^ The medication management service in the RACF is multidisciplinary, with the involvement of external health professionals (general practitioners [GPs] who prescribe medicine and community pharmacists who dispense the medicine) and internal staff (nurses and/or credentialed aged care workers who administer the medicine).

### 2.2 Participants and recruitment

The research team engaged with each RACF in advance of the EMBRACE randomised controlled trial that aims to evaluate different guideline implementation strategies.^25^ Participants were invited through the RACFs using convenience sampling to achieve maximum variation. The interviewer introduced themselves to the participants and provided a brief explanation of the project, confidentiality, withdrawal and data use. Each participating organisation was provided with remuneration in recognition of staff time for participation as a part of their engagement in a wider trial.^25^ Residents, family and informal carers, and health professionals external to the facility received an AUD$50 gift voucher in recognition of their time.

### 2.3 Data collection

Data were collected via semi‐structured interviews (~30 min) conducted on Zoom or Microsoft Teams. The interview guide (Appendix S1) developed by the research team explored the people, processes and infrastructure within the facility and broader organisation to ensure the appropriate use of psychotropic medications. The interviews sought to examine the strengths and challenges of current approaches and to identify specific enablers and barriers to implementing the new Psychotropic Guidelines. The interview guide was reviewed by two health professionals with expertise in psychotropic medication use in aged care. The interviews were conducted until thematic saturation by two authors (ALC, SS) with experience in qualitative research and the care of older people with dementia or cognitive impairment; audio‐ and/or video‐recorded and transcribed verbatim by a professional transcription service. Participants were provided with a summary of the interview and an opportunity to clarify or correct it.

### 2.4 Data analysis

The interview transcripts were independently coded by two authors (ALC, SS) using the NVivo Qualitative Data Analysis Software v.1.6.1 (QSR International, USA) (Appendix S2), who regularly discussed the codes and ensured consistency, and the entire team discussed the preliminary findings. Deductive thematic analysis^26^ was performed using the CFIR, which consists of five overarching domains (innovation, outer setting, inner setting, individuals and implementation process).

In this study, the CFIR innovation was the new Psychotropic Guidelines,^7^ which includes 15 recommendations and 49 good practice statements related to the appropriate use of psychotropic medications.^27^ Interview data were analysed to identify contextual factors related to three CFIR domains—outer setting (external context of implementation), inner setting (organisational context of implementation) and individuals (people associated with organisation for implementation). The identified contextual factors informed the CFIR implementation process domain (guideline implementation strategies) elaborated in the discussion. These strategies were iteratively developed by two authors (SS, ALC) through cod modifiable contextual factors important nsensus and agreed upon by the research team.

### 2.5 Ethics

This study was approved by the University of Queensland Human Research Ethics Committee—HABS LNR (2022/HE001147). Prior to conducting the study, approval was sought from each participating aged care provider organisation, and written consent was obtained from each participant.

## 3 RESULTS

There were 33 participants (Table 1)—28 health‐care professionals (11 registered nurses, eight pharmacists, four nurse practitioners, two occupational therapists, two GPs and a geriatrician) and five consumers (two residents and three resident family members).

**TABLE 1:** Interview participants and their roles across the participating organisations.

Participants (*n* = 33)	Roles	*n* (%)
Staff of aged care provider organisations (*n* = 18)	Manager	6 (18)
	Nurse Practitioner	4 (12)
	Care Coordinator	2 (6)
	Research and Education	2 (6)
	Others^a^	4 (12)
Visiting health professionals external to aged care provider organisations (*n* = 10)	RMMR and QUM Pharmacist	5 (15)
	Pharmacy Director or Clinical Governance	2 (6)
	General Practitioner or Geriatrician	3 (9)
Consumers (*n* = 5)	Resident	2 (6)
	Resident Family Member	3 (9)

*Note*: The overall % do not add up to 100 due to rounding.

Abbreviations: QUM, Quality Use of Medicines; RMMR, Residential Medication Management Review.

^a^ Others: clinical governance, behavioral management, and pharmacy service.

### 3.1 Factors influencing the implementation of the new Psychotropic Guidelines in Australia

#### 3.1.1 Outer setting domain

**FIGURE 1:**
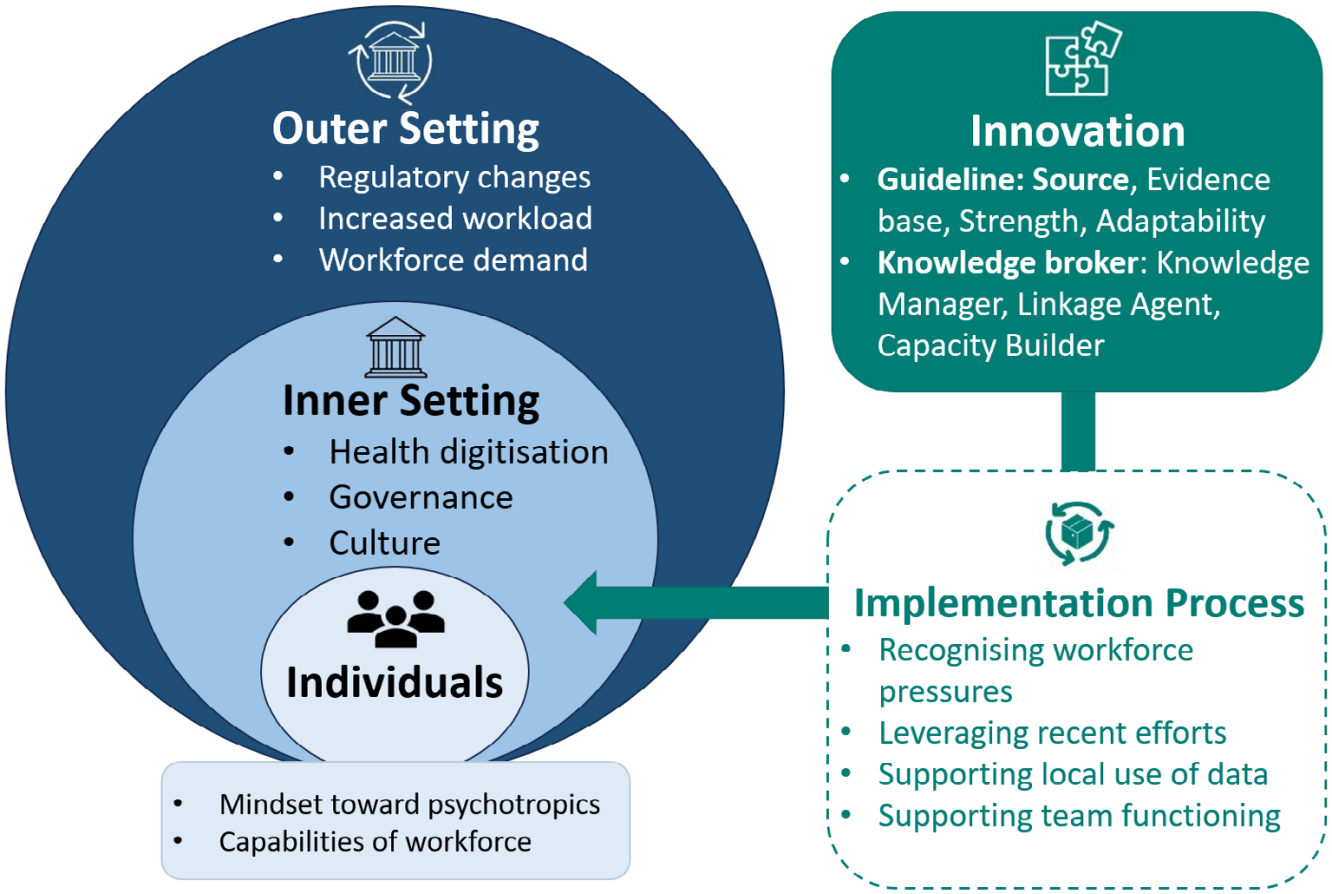
Consolidated Framework for Implementation Research on Australia's new Psychotropic Guidelines (adapted from Damschrodner et al., 2022^21^).

Three outer setting factors were identified: regulatory changes, increased workload and workforce demand (Figure 1, Table 2).

**TABLE 2:** Outer setting domain factors influencing the implementation of new Psychotropic Guidelines and illustrative quotes.

Factors	Illustrative quotes
Regulatory changes	
Undertaken considerable amount of work to comply with regulatory changes	[Q1] – …our behaviour support plan…added to that was developing this register to monitor who is at high risk, who could be weaned off any restricted practices…it was a huge amount of changes. *[0205‐Occupational therapist‐Behaviour support lead and dementia consultant]*
	[Q2] – This is a requirement from the Age Care Quality Commission…to make sure that with any psychotropic medication, we either have a valid diagnosis for the condition or we have a valid consent from the family and the GP [*General Practitioner*] and a restricted practice form completed prior to administering any medication. *[0406S2‐Nurse‐Service manager]*
Conflict arising from shift in responsibilities on restrictive practice	[Q3] – We still have a lot of […] GPs that would push back a lot of those consultations to us as RNs and even managers, “You go talk to the family,” when in fact the legislation requires them to do it. So not a lot of the GPs are doing it and even if they say they are. *[0207‐Nurse‐Care manager]*
	[Q4] – When I chart the psychotropic, certainly, the thing comes up to say, “Have you got consent for this from anyone?” And a lot of the time, it's a simple– for me, I'm time‐poor…So the consent often for me is a text to the next of kin. Now, I understand that that's not often good enough.…… And do you have to go to [Tribunal] and all of that, right? Again, it is beyond the scope of my comprehension. *[0209‐General practitioner]*
	[Q5] – Our last Commission visit, there are a couple where the GP's had said that a certain medication wasn't a chemical restraint when realistically it actually was. Anything that changes their behaviour or has the potential to change behaviour is a chemical restraint. *[0302‐Nurse‐Clinical nurse manager]*
Streamlined psychotropic medication use process	[Q6] – …making sure that when we were using psychotropics, they were an absolute last resort option. So making sure that people had a behavioural support plan in place, that behaviour support strategies were trialled, and then psychotropics were considered. And then in the context of that, once we've been involved with the resident, we sort of oversee the psychotropic use. And the DCMs [*Dementia Care Manager*] …check that all the regular monitoring and stuff is happening. *[0201‐Occupational therapist‐Dementia consultant]*
Facilitated psychotropic medication review	[Q7] – I think the best thing about this change has been putting in place processes that ensure that we are reviewing the psychotropic medication for cessation, and that they're not being put on this psychotropic for the rest of their life. *[0205‐Occupational therapist‐Behaviour support lead and dementia consultant]*
Improved accountability and communication	[Q8] – …the GPs are very very cautious when it comes to charting psychotropics. It's essentially antipsychotics … So even if it's clinically needed and that person is very very agitated and aggressive, they're more cautious in charting this medication. *[0202‐Nurse practitioner‐Care Manager]*
	[Q9] – The communication between sites and the clinical excellence team and the governance team has improved significantly. *[0302‐Nurse‐Clinical Nurse Manager]*
Increased workload associated with quality use of psychotropics	
Compliance focused	[Q10] – …it is counted as psychotropic, and it clearly says that it has side effects. So we are just going to go with what we decided…We just don't want any noncompliance when it comes to that point of view, so we decided to just go with it… *[0204S2‐Nurse practitioner‐Clinical governance lead]*
Definition of psychotropic medication broadly applied	[Q11] – I think I see a lot of frustration sometimes with RNs [*Registered Nurses*] on the floor and even with the CNS [*Clinical Nurse Specialist*] and that's across the different units I've worked with, with the amount of medications that's included on the list that people have to sort of follow up because it is a broad list. When you say psychotropic it can get tricky, and sometimes the basic ones like for anti‐nausea is included…I do understand the clinical background of it and when you explain it to them, of course, it is logical. *[0207‐Nurse‐Care manager]*
	[Q12] – I'm telling you, “Okay. Fine. Pregabalin, antidepressants, antipsychotics, mood stabilizers? Granted, right? I go with it, right? Opioids. Completely different group, but also highly, highly granted. But Maxolon [*metoclopramide*], Stemetil [*prochlorperazine*], Motilium [*domperidone*] – what else? – PRN [*when required*] in case they vomit. So, I have to validate the Maxolon [*metoclopramide*] every 3 months, but write the reason. *[0209‐General practitioner]*
Managing workload and compliance pressure	[Q13] – there's extra work…we do [inaudible] monthly reporting is what the national quality indicator look for. But it's not. They have an entirely different definition of report what they need. So, on the top of the reports that we do, like self‐assessment tool, we have monthly–we save all of that. And I have to do the number of psychotropics; how many came in restraint. I do all of that as part of our monthly routine reports. *[0204S2‐Nurse practitioner‐Clinical governance lead]*
	[Q14] – We currently are actually doubling up on consent. Not only with the four forms for the chemical restraints, but the prescribers– getting them to contact the next of kins– …there wasn't really much guidance as to whether we provided that to the nurse practitioner. Or the GP or just let them know that, “Hey, we've let the consumer rep know that this is what it is and the benefits and the risks.” And [leadership staff] said, “Oh, well, actually, you nurses aren't meant to be doing that.” So, it's very confusing. *[0302‐ Nurse‐Clinical nurse manager]*
	[Q15] – So, along with them falling in line and really trying to review them, there is this level of anxiety as to what indications don't I have to review three monthly? … They sent me a note, “Please, XXX. Not sure on risperidone,” and they actually had sodium valproate. *[0304‐RMMR and QUM pharmacist]*
Workforce demand	
High staff turnover and workforce shortage	[Q16] – So we've got one clinician supporting high turnover of leadership roles… We're not getting leaders who are traditionally from aged care. So, we're getting leaders from health. So, their understanding, they need a fair bit of support to come into this aged care world. So that's our main remit at the moment is orientation with our limited resources with the high turnover. People are just coming and going very, very quickly in those leadership roles. *[0301‐Nurse‐Manager clinical excellence]*
	[Q17] – I mean, we've got shortages of RNs, so they're filling positions with people that have got very limited experience that no longer have that clinical experience that RNs of the past had where you had to go and work in a hospital. They had short stints in different areas, and they don't really have that overall thing. *[0208‐RMMR and QUM Pharmacist]*
Understaffing	[Q18] – …but they [nursing staff] are also in a situation where they are under enormous pressure. They're understaffed because of funding, they are human beings, and you get people arcing up and playing up and you haven't got the staff to cope with it. We have to understand that there may be a reason that they just ask for some medicines to quiet someone down because they're feeling really stressed themselves and they're not coping with it. I think we just have to keep that in mind because it's been underfunded for a long, long time. *[0304‐RMMR and QUM pharmacist]*

##### Regulatory changes

Participants reported that aged care organisations have *undertaken a considerable amount of work to comply with regulatory changes* following the Royal Commission [Q1, Q2]. Key work included the implementation of a psychotropic register for collecting data on psychotropic medication, behavioural support plans for restrictive practices and provider responsibilities to ensure behavioural support plans and consent for any restrictive practice [Q1, Q2]. Participating staff at aged care organisations described a clearer recognition of their responsibilities in ensuring consent and the documentation of behavioural support plans, which has led to some conflicts between prescribers and aged care organisation staff in terms of what is considered a restrictive practice. Where these *conflicts have arisen*, staff at aged care organisations have taken *responsibility* for determining whether a restrictive practice was in place and ensuring appropriate documentation [Q3–Q5]. Overall, most participants believed that changes positively impacted the care of residents, as they *streamlined the process for psychotropic medication use* [Q6], *facilitated psychotropic medication review* [Q7], *improved accountability* [Q8] and *improved communication* [Q9].

##### Increased workload associated with quality use of psychotropics

An interview with a clinical governance lead (nurse practitioner) suggested that ensuring regulatory *compliance* was a key driver of decision‐making in relation to changes in procedures for psychotropic medication use [Q10]. A care manager and a GP highlighted that the *definition of psychotropic medication for regulatory purposes is applied broadly* [Q11, Q12]; consequently, a lot of medications are being considered for the psychotropic register, and this has contributed to the existing workload. The interviews with nursing staff and a pharmacist highlighted that their capacity to *manage the workload and compliance pressure* alongside other clinical responsibilities is limited [Q13–Q15].

##### Workforce demand

A nurse and a pharmacist highlighted that the demand for a skilled aged care workforce continues to remain high due to *high staff turnover and workforce shortages* [Q16, Q17], which can have a greater impact on guideline implementation. A pharmacist explicitly stated that due to *shortages* of registered nurses in RACFs, their positions are being filled by new staff with limited clinical experience [Q17]. Another pharmacist described pressure on aged care staff due to *understaffing issues* and believed that this pressure might be a consequence of underfunding [Q18].

#### 3.1.2 Inner setting domain

Three major inner‐setting factors were identified: health digitalisation, governance and compliance culture (Figure 1, Table 3).

**TABLE 3:** Inner setting domain factors influencing the implementation of new Psychotropic Guidelines and illustrative quotes.

Factors	Illustrative quotes
Health digitalisation	
Using a range of different software providers	[Q19] – what they're asking from the psychotropic register, what they want, the information that they want is very in‐depth, and not all of it I can populate from [*software 1*]. So, I do have to go into other programs to find that information. So, it's not a cohesive system yet, if you understand. *[0102‐Nurse‐In‐charge]*
	[Q20] – they've all transitioned to [*software 2*], which is a full electronic system. So that's taken out a lot of the little issues that used to be before. There are still occasional things that I'll find where the doctor in the electronic system hasn't actually written in the complete directions. [inaudible] entered it into the system, so the system packs it, but they missed out on directions. *[0208‐RMMR and QUM pharmacist]*
	[Q21] – we were trying to record the medications on a centralized– on a reporting system within [*software 3*]. Within the client system. So, after you'd done the bits and pieces, you'd type in the medication and whether it was a restraint or not and when it was going to be reviewed and consent. *[0301‐Nurse‐Manager clinical excellence]*
	[Q22] – But we've never had anything electronic. We just rely on an Excel spreadsheet to pump all the information into– and it's very manual for the girls. They use [*software 4*], which doesn't have a medication system. So, they've got to go through all the medication charts independently and add that into the Excel spreadsheets and submit all the data and then get our reports back. *[0402‐Nurse‐Quality and education support]*
Using a manual system	[Q23] – But then we found the other systems issue was that you have to manually archive all of those documents. So, they weren't being archived. So, the register was never up‐to‐date, and it took a whole heap of time in trying to maintain this system. So then again, we went to an Excel document. *[0301‐Nurse‐Manager clinical excellence]*
	[Q24] – The drug usage reports are quite manual, and it takes 2 or 3 days to get them out to every facility, even though they're sort of scheduled. So that's a challenge. It's a big challenge. *[0503‐Pharmacist‐Clincial governance advisor]*
Governance	
Quality and compliance team	[Q25] – In terms of like now ongoing changes, we brought a whole quality and compliance team that are in very close connection with whatever legislative requirements are going on. And they will communicate with the rest of our organization about these changes, mostly to the clinical team. *[0205‐Occupational therapist‐Behaviour support lead and dementia consultant]*
Additional clinical leads	[Q26] – So we're just hiring a couple of more clinical leads now. And we're hoping to finally do kind of a role where the clinical leads are delegated to provide support for three to four sites maximum … *[0301‐Nurse‐Manager clinical excellence]*
Dementia consultant and behavioural support lead	[Q27] – So, yeah, we are a unique organization in that we have a dementia consultant and a behavioural support lead to make sure that those processes are carried out… And for an external validation, if we're not sure and we want another eye on things, we'll refer to Dementia Support Australia. *[0201‐Occupational therapist‐dementia consultant]*
Communication channels	[Q28] – So we update it according to that and we also discuss it in our three‐monthly MAC [Medication Advisory Committee] meetings with the pharmacy as well, our ratios of psychotropics and polypharmacy and all that key areas. *[0406S2‐Nurse‐Service manager]*
	[Q29] – Look, I think one of the things that I do with my residents – and I can't say for other GPs – is at least once a year, I have a case conference. *[0209‐General Practitioner]*
Poor engagement with prescriber	[Q30] – Finding it really hard to get a hold of the doctors …I would call the doctors and sometimes they don't have the time to chat to me, so I'll have to email them. And sometimes I don't hear a response. *[0101‐Pharmacist‐onsite pharmacy service]*
Culture	
Data collection	[Q31] – And then when you actually present data back to them, then it make them think about, “Okay, why is our average too high? So, what happened? What can we do?” So, make them to think rather than just– if we don't have those quality indicators, then they don't know where they are sitting at. So yeah. *[0502‐RMMR and QUM pharmacist]*
	[Q32] – Yes, it does actually go both ways, where we can see how we've managed certain behaviours or sort of behaviours of concern with medication, how– we've improved that, which results in an improved quality of life as a resident, that type of thing. *[0402‐Nurse‐Quality and education support]*
Recipient‐centeredness	[Q33] – So we are an ageing in place facility, and we use a home‐to‐home method. And the home‐to‐home is basically they leave their home and they move to our home, which becomes their home. So, our care staff do everything with the residents, and we call them housemates. So, they're like friends that are living with the resident. And it's always the same housemates in the same area. The housemate does their medication. The housemate does their personal care. The housemate serves them their meals, so it's really like a friend being there to help them. *[0102‐ Nurse‐In‐charge]*
	[Q34] – So it's a good idea to find the resident's picture, whole picture of what they were, how have they lived all their life, and a little bit of their hobbies and what they used to love in the past. And then we can use that strategies to manage their behaviour, rather than any chemical, any pharmacological options. *[0406S2‐Nurse‐Service manager]*
Staff support	[Q35] – So, if they do education, they do it more on a– that's an education day for RNs, and they just take all the RNs off the floor, and then you might do education. *[0208‐RMMR and QUM pharmacist]*

##### Health digitalisation

Aged care senior staff and a pharmacist indicated an ongoing effort in developing a digital health system for improving the functional performance of day‐to‐day activities and that each organisation was increasingly transitioning to a digital health system using *a range of different software providers* [Q19–Q22]. At the time of the interviews, no organisation's digital system automatically collected all the data related to the quality use of psychotropics; *manual approaches* were required to generate drug usage reports [Q23, Q24].

##### Governance

Aged care staff suggested that their organisations had strong clinical governance that was further strengthened by setting up new *quality and compliance teams* [Q25], hiring *new clinical leads* [Q26] and introducing newer roles such as *dementia consultants and behavioural support leads* for the behavioural management of dementia [Q27]. They also mentioned that *communication* within the organisation was mainly channelled through formal/informal meetings such as quarterly *Medication Advisory Committee meetings* [Q28] for medication management and annual case conferences in *Multidisciplinary Team meetings* [Q29] for formal review and communication with residents and their family member(s) at various levels. An onsite pharmacist stated that despite a good communication system, the *engagement of internal staff with prescribers* was a challenge [Q30].

##### Compliance culture

A pharmacist and a nurse suggested an increasing *culture of data collection* from practice with a focus on psychotropic medication and restrictive practices, mainly to meet regulatory requirements and to gain knowledge about the current use of psychotropics and restrictive practices [Q31, Q32]. These data were mainly used at the individual level rather than the system/facility level [Q32]. Participants who were internal staff expressed a culture of *recipient‐centeredness* in the delivery of resident care, such as prioritising their needs and providing a holistic approach to behavioural management [Q33, Q34], which could facilitate the implementation of the new Psychotropic Guidelines. A participant who was a pharmacist suggested that aged care organisations provide *support staff* education related to psychotropic medication and behavioural management [Q35]. This culture could be important for guideline implementation.

#### 3.1.3 Individual domain

Two main factors related to individuals were identified: mindset toward psychotropics and workforce capabilities (Figure 1, Table 4).

**TABLE 4:** Individual domain factors influencing the implementation of new Psychotropic Guidelines and illustrative quotes.

Factors	Illustrative quotes
Mindset towards psychotropics	
Individualised clinical decision‐making	[Q36] – I predominantly am patient care driven… Because we don't like seeing residents in distress and our primary—certainly, my primary goal is that people are allowed to age with dignity and comfort, right? *[0209‐General practitioner]*
Compliance and individualised care	[Q37] – We really want to have great outcomes for clients at the end of the day. And we want to be compliant. *[0301‐Nurse‐Manager clinical excellence]*
Compliance focused	[Q38] – We do the reports on all psychotropic. If that's what they want, it would be so much easier because we do it on a monthly. So, this NQI will come as an extra work because they don't want any of this. They want something separate. And so that kind of affects the work when it comes to this reporting. That's when it becomes so focused on compliance because we have to make sure we do a monthly thing, and we have to make sure we do the NQI thing. *[0204S2‐Nurse practitioner‐Clinical governance lead]*
Pharmacological management of changed behaviour	[Q39] – And the risk that [the resident] takes of an adverse […] event is worth it for the benefit … I mean, I'm not saying it's worth [for the resident] getting [an adverse event], but with him suffering so greatly from anxiety and paranoia, it would be– and if you could treat that in a relatively effective way, it was worth it for him because his quality of life was terrible. *[0210‐Family member of resident]*
	[Q40] – And from the moment she started taking those medications, it did put her in a better place. The anxiety was extreme, and she would often leave our house… Without the medication, I think the psychotic episodes were getting worse. *[0211‐Family member of resident]*
Workforce capabilities	
Skills	[Q41] – you have to depend on your stats and then you have to look into the time and then put interventions there and then—and I also did the Plan‐Do‐Check‐Act cycle, PDSA [*Plan, Do, Study, Act*] cycle to see when I do the interventions, is that working after a few weeks? And that is very useful in larger setting where you can't do multiple thing at one—because if you are doing multiple things at one step in a large facility, it's going to set you to failure. So just going with your stats, the more priority, yeah, you can divide your priority base and then start your action. *[0404‐Nurse‐Care Coordinator and Dementia Consultant]*

##### Mindset toward psychotropics

Health professional participants expressed varying mindsets towards psychotropic medication use. A GP approached psychotropics within an individual clinical context and worked towards the goal of determining whether an individual benefited from psychotropic medication use (*individualised clinical decision‐making mindset*) [Q36]. A clinical excellence manager (nurse) suggested a similar approach but generally with a strong compliance focus [Q37]. A nurse practitioner had felt that psychotropics were almost always inappropriate and worked towards the goal of reducing use as much as possible (*compliance‐focused mindset*) [Q38]. Family members of residents with dementia expressed support for *pharmacological management of changed behaviors* if it benefited the resident in terms of mitigating psychotic symptoms and improving quality of life [Q39, Q40].

##### Workforce capabilities

The interviews suggested that aged care staff generally have *skills* for measuring, analysing, developing and evaluating changes related to psychotropic medication use. For instance, a dementia consultant (also a nurse coordinator) had used psychotropic medication data in specialist dementia units and residential aged care to identify areas for improvement and put in strategies to improve care and assess outcomes [Q41]. However, there was limited evidence that the full cycle of continuous quality improvement is embedded within day‐to‐day processes.

## 4 DISCUSSION

Key interconnected and modifiable contextual factors important for implementing the new Psychotropic Guidelines were related to the workforce, digitalisation, governance, culture and mindset towards psychotropics. Together, these factors suggested four key implementation strategies for guideline implementation: (i) recognising workforce pressure within organisations, (ii) leveraging the recent efforts in aged care organisations, (iii) supporting organisations in the local use of data and (iv) supporting team functioning.

### 4.1 Recognising workforce pressures within the organisations

Australian aged care organisations have undergone a major shift in the contexts of people, process and infrastructure. Our study found that these changes had workforce implications. Previous research has suggested that workforce factors such as staff shortages, high staff turnover, workload pressure and competing priorities are barriers to the implementation of complex interventions and clinical practice guidelines in long‐term care^28^, ^29^ and a variety of settings,^30^ respectively. Workforce issues can also mitigate against the use of behavioural management as an alternative to psychotropics. An implementation strategy that aligns the guideline implementation with existing workflows and, where possible, a reduced workload for key staff could be beneficial. A reduced staff workload may arise by ensuring that implementation initiatives are targeted, utilising digitalisation for automation, and supporting team functioning. Implementation can also be aided by engaging with RACF leaders, local champions and family members. Studies have shown that engaging with leaders and champions can facilitate the implementation of complex interventions in long‐term care and nursing homes.^29^, ^31^ Engaging implementation facilitators in the planning and execution of implementation processes could provide ownership and assurance, promote receptivity, enhance satisfaction and improve self‐esteem related to guideline implementation.^32^ Additionally, adequate and clear communication within an organisation has been found to facilitate the implementation of clinical practice guidelines.^30^ We found that the participating RACFs already had a strong in‐house communication systems, which could be used to facilitate stakeholder engagement. Incorporating knowledge brokers to support guideline implementation presents a further opportunity for improved communication The evidence suggests that knowledge brokers as linkage agents, capacity builders and knowledge managers have a significant impact on the implementation of clinical practice guidelines.^33^, ^34^

### 4.2 Leveraging recent efforts in aged care organisations

Our study identified several activities undertaken by aged care organisations that could be leveraged to support and influence the implementation of the new Psychotropic Guidelines, including the ongoing development of health digitalisation, the strengthening of governance and the development of a culture of data collection. The Royal Commission identified several measures for improving care standards in Australian aged care organisations, including the adoption of digital technology such as electronic medication management,^35^ increasing nursing time and having aged care on‐site pharmacists. To support medication management, the Australian Government allocated grant funding to residential aged care for electronic National Residential Medication Charts products.^36^, ^37^ The appropriate use of technology and integrated information systems could facilitate the implementation of clinical practice guidelines.^30^ The ongoing development of health digitalisation, coupled with existing strong clinical governance, could be utilised to monitor the effectiveness of guideline implementation and provide improved visibility and oversight of psychotropic medication. Evidence suggests that digital dashboard technology, using quality indicators, can support the identification of patients at risk of inappropriate use of psychotropic medication in long‐term care.^38^ The development of digital health could also serve as a single platform for improved communication between the internal and external health‐care providers of care in aged care organisations, which is a current need and challenge in aged care settings. This platform could be utilised for better engagement and to disseminate information about the new Psychotropic Guidelines. Our study highlighted that digital infrastructure development and the use of software were not uniform across the organisations. Therefore, it is important to understand the specific context of an organisation's digital infrastructure development and tailor the strategy for guideline implementation accordingly. The Australian government has also mandated the introduction of 24/7 registered nurses and increased minimum nursing care minutes in RACFs^39^ as well as funding to employ credentialed pharmacists to work on‐site in RACFs in a clinical role.^40^ These changes provide opportunities to support the effective implementation of the new Psychotropic Guidelines.

### 4.3 Supporting organisations on local use of data

Our study identified an increasing capacity for data collection in aged care organisations. In terms of psychotropic medications, the primary objective was to meet regulatory requirements; however, there are further opportunities for utilising these data to gain knowledge to inform practice change. Supporting organisations to use local data for continuous quality improvement is an important strategy for the effective implementation of the new Psychotropic Guidelines. This strategy can also strengthen the learning health system processes in organisations.^41^, ^42^ Learning health systems have shown health benefits irrespective of setting and geography^43^ and have allowed improved transition of innovation into practice.^44^ The routinely collected local data could be iteratively utilised to identify gaps in practice and benchmarking facilities, subsequently informing local action plans for necessary quality improvements. Existing infrastructure for psychotropic medication‐related data collection could be utilised, and indicators established based on the data, which is being trialled in an ongoing clinical trial.^25^ This would require staff training and time allocation, and support for data analysis and interpretation. Customised reporting tools or dashboards could be developed to easily access and visualise the data at the local level.

### 4.4 Support team functioning

Challenges related to engaging external health professionals, conflicts arising in relation to shifting regulatory responsibilities and high staff turnover were also identified during this study. Building and supporting effective interdisciplinary teams and engaging external health professionals in clinical governance processes is an important strategy for implementing the new Psychotropic Guidelines. Interdisciplinary interventions in RACFs have shown a positive impact on resident outcomes including but not limited to a reduction in psychotropic medication use and restraint,^45^ with the most successful interventions involving physicians and/or pharmacists and supported by team communication and coordination. Indeed, the key features of a successful interdisciplinary team approach are collaboration, shared leadership, and shared decision making between members of different disciplines and consumers (patient and family).^46^ This provides strong support for the knowledge broker role functioning as a linkage agent and a capacity builder in improving the team functionality and cohesiveness. Strategically utilising the knowledge brokers to effectively link stakeholders could ensure that guidelines are uniformly applied across the organisation and that prescribers are optimally engaged. This is much needed because our findings indicate a varying perception of participants on psychotropic medication use. In addition, GPs and geriatricians in Australian RACF provide their services as visiting health professionals external to the organisations.^47^ Strategies to ensure prescribers are engaged as a part of the team and that the team supports best practice management of dementia are needed. Besides linking the team, a knowledge broker can support team functioning through the capacity builder role, providing tailored education and training support. This role is important given our findings that organisations frequently need to train new staff due to high staff turnover. The knowledge brokers will need to work closely with organisational leaders to identify staff who need training for successful guideline implementation.^48^ The knowledge broker's ability to engage and build capacity within the organisations could be useful in resolving some of the conflict arising due to changes in regulatory responsibilities on restrictive practices. The comprehensive onboarding of new staff and continuing to identify and address knowledge gaps is important for ensuring that all staff members have the capability to support appropriate psychotropic use.

### 4.5 Strengths and limitations

Participants were from diverse backgrounds, including those in leadership positions, clinicians, residents and family members. Participants had good knowledge of the people, processes and infrastructure needed to reform psychotropic medication use. The participants worked with one of four independent not‐for‐profit aged care provider organisations from four Australian states. It is possible that the findings may not be generalisable to the experience of healthcare professionals working with other aged care provider organisations (e.g. private, public‐sector). While it was advantageous to compare and contrast the facilitators and barriers to implementation across the four different aged care organisations, different aged care organisations may have implemented other initiatives in response to recent regulatory changes and therefore considering the local context might add extra value. The CFIR framework was used on pre‐implementation interview data from key stakeholders of aged care provider organisations. Therefore, the findings should be interpreted within this context. Strategies for the guideline implementation process are also useful for continuous quality improvement in aged care and may require policy support for their effectiveness.

## 5 CONCLUSIONS

The Australian aged care contextual factors influencing the implementation of the new Psychotropic Guidelines relate mainly to regulatory change, workforce pressure, organisational efforts and individual qualities. These factors are generally interconnected and strategically modifiable. Balancing regulatory compliance and aged care workload could be achieved by leveraging organisational efforts for health digitalisation, clinical governance, communication and data utilisation within organisations. Uniform adherence to guidelines and effective interdisciplinary collaboration could be ensured by supporting team functioning and engaging external health professionals.

## FUNDING INFORMATION

This study was supported by the Australian Government Medical Research Future Fund (GA187306). AJC was supported by an NHMRC Emerging Leadership 1 Grant (APP2009633).

## CONFLICT OF INTEREST STATEMENT

JSB has received grant funding or consulting funds from the National Health and Medical Research Council, Medical Research Future Fund, Victorian Government Department of Health and Human Services, Dementia Australia Research Foundation, Yulgilbar Foundation, Aged Care Quality and Safety Commission, Dementia Centre for Research Collaboration, Pharmaceutical Society of Australia, Society of Hospital Pharmacists of Australia, GlaxoSmithKline Supported Studies Programme, Amgen, and several aged care provider organisations unrelated to this work. All grants and consulting funds were paid to the employing institution. AJC has received grant funding or consulting funds from the Medical Research Future Fund, Dementia Australia Research Foundation, and Pharmaceutical Society of Australia. All grants and consulting funds were paid to the employing institution. The other authors declare that they have no competing interests.

## Supporting information


Appendices S1–S2

## Data Availability

All data relevant to the study are included in the article.
