# Supplementary material for: Implementing Australia's new guidelines for appropriate psychotropic medication use in residential aged care: Applying the Consolidated Framework For Implementation Research
Source: Australas J Ageing. 2025 Mar 14;44(1):e70013. doi: 10.1111/ajag.70013 (PMC11907677; doi:10.1111/ajag.70013)
Supplement: Supplementary file 1 — Appendices S1–S2 [file AJAG-44-0-s001.docx]

**Appendix 1**

**Interview guide**

The guide will be adapted for semi-structured interviews. Additional prompts may be added in response to themes that are developed throughout the analysis.

The focus of the interviews will differ between healthcare providers/facility staff and residents/carers.

**Care providers and facility staff**

***Introduction***

Welcome. Thank you for participating in this project.

The aim of the study is to examine quality improvement processes in relation to medication management within the facility.

A key focus of the study is specifically regarding quality improvement processes regarding psychotropic medicines. These medicines are used to help sleep, improve mood and to help manage changed behaviours in dementia (e.g. benzodiazepines, antidepressants and antipsychotics). We would like to understand the people, processes and infrastructure that are available to support the safe and effective use of psychotropics.

We thank you for sharing your views. The information you provide will remain confidential. The interview will be recorded.

All identifying information will be stored securely and only be available to the researchers. All participants will de-identified in the transcripts. A short summary of the key themes discussed in the interview will be emailed to you and you will have an opportunity to comment on the summary.

***Discussion prompts***

Introduction.

Please describe your current role

What roles do you currently have with the use or monitoring of psychotropics?

Current processes for continuous quality improvement of psychotropics

Please describe any facility/group-level approaches are used to ensure safe and effective use of psychotropics?

Who is involved?

How is the approach supported?

How does change happen? (Who enacts change? How is this facilitated?)

Can you provide an example?

What do you see as the strengths of the current approaches?

What do you see as the challenges of the current approaches?

[What would you ‘start, stop, continue’ in relation to current continuous quality improvement processes for medicines/psychotropics?]

Best practice and quality improvement process are changing in relation to use of psychotropics in aged care

What do you see as the key changes that are happening?

[Examples: inclusion of psychotropic use among mandatory quality indicator program; best-practice recommendations in terms of prescribing and monitoring psychotropic use (e.g. DCRC NHMRC guideline)]

[Use components of DCRC NHMRC guideline as prompts where appropriate; an incomplete list of examples: resident consent for psychotropics (GPS2, GPS5); decision-making regarding initiation (GPS13), monitoring (GPS16)]

How well do you think existing processes can accommodate these changes?

What additional processes or infrastructure will be necessary to implement these changes?

What are the facilitators of change?

What are the barriers to change?

Do you have any examples of proposed changes that you think will be difficult/easy to implement?

Is there anything else you would like to share regarding continuous quality improvement systems and processes in relation to psychotropic medicines?

**Appendix 2**

**Coding scheme**

Deductive codes for Consolidated Framework for Implementation Research (CFIR)

| **CFIR domains** | **Codes and sub-codes** | |
| --- | --- | --- |
| Innovation | Guideline | Source |
|  |  | Evidence |
|  |  | Strength base |
|  |  | Adaptability |
|  | Knowledge broker | Knowledge manager |
|  |  | Linkage agent |
|  |  | Capacity builder |
| Outer setting | Regulatory changes | Undertaken considerable amount of work |
|  |  | Conflict arising from shift in responsibilities on restrictive practice |
|  |  | Streamlined psychotropic medication use process |
|  |  | Facilitated psychotropic medication review |
|  |  | Improved accountability and communication |
|  | Increased workload associated with quality use of psychotropics | Compliance focused |
|  |  | Definition of psychotropic medication broadly applied |
|  |  | Managing workload and compliance pressure |
|  | Workforce demand | High staff turnover and workforce shortage |
|  |  | Under staff |
| Inner setting | Health digitalisation | Using a range of different software providers |
|  |  | Using a manual system |
|  | Governance | Quality and compliance team |
|  |  | Additional clinical leads |
|  |  | Dementia consultant and behavioural support lead |
|  |  | Communication channels |
|  |  | Poor engagement with prescriber |
|  | Culture | Data collection |
|  |  | Recipient-centredness |
|  |  | Staff support |
| Individuals | Mindset towards psychotropics | Individualised clinical decision-making |
|  |  | Compliance and individualised care |
|  |  | Compliance focused |
|  |  | Pharmacological management of changed behaviour |
|  | Capabilities of workforce | Skills |
